# High‐Frequency Variability of Small‐Particle Carbon Export Flux in the Northeast Atlantic

**DOI:** 10.1029/2018GB005963

**Published:** 2018-12-18

**Authors:** Roséanne Bol, Stephanie A. Henson, Anna Rumyantseva, Nathan Briggs

**Affiliations:** ^1^ School of Ocean and Earth Sciences University of Southampton Southampton UK; ^2^ Now at NIOZ Royal Netherlands Institute for Sea Research Texel The Netherlands; ^3^ Now at Department of Earth Sciences Utrecht University Utrecht The Netherlands; ^4^ National Oceanography Centre Southampton UK

**Keywords:** carbon export, mixed‐layer pump, transfer efficiency, optical backscatter, gliders

## Abstract

The biological carbon pump exports carbon fixed by photosynthesis out of the surface ocean and transfers it to the deep, mostly in the form of sinking particles. Despite the importance of the pump in regulating the air‐sea CO_2_ balance, the magnitude of global carbon export remains unclear, as do its controlling mechanisms. A possible sinking flux of carbon to the mesopelagic zone may be via the mixed‐layer pump: a seasonal net detrainment of particulate organic carbon (POC)‐rich surface waters, caused by sequential deepening and shoaling of the mixed layer. In this study, we present a full year of daily small‐particle POC concentrations derived from glider optical backscatter data, to study export variability at the Porcupine Abyssal Plain (PAP) sustained observatory in the Northeast Atlantic. We observe a strong seasonality in small‐particle transfer efficiency, with a maximum in winter and early spring. By calculating daily POC export driven by mixed‐layer variations, we find that the mixed‐layer pump supplies an annual flux of at least 3.0 ± 0.9 g POC·m^−2^·year^−1^ to the mesopelagic zone, contributing between 5% and 25% of the total annual export flux and likely contributing to closing a gap in the mesopelagic carbon budget found by other studies. These are, to our best knowledge, the first high‐frequency observations of export variability over the course of a full year. Our results support the deployment of bio‐optical sensors on gliders to improve our understanding of the ocean carbon cycle on temporal scales from daily to annual.

## Key Points

- Gliders captured a full annual cycle of high‐frequency POC variability over the upper mesopelagic zone
- Small‐particle transfer efficiency displayed a strong seasonality, peaking in winter and early spring
- The data indicate a significant annual carbon supply to the mesopelagic zone by small particles through the mixed‐layer pump

## 1 Introduction

The ocean is an important sink for CO_2_ through the combined effect of the solubility pump and the biological carbon pump (Sabine et al., 2004) and has taken up about 26% of all anthropogenic emissions since the industrial revolution (Le Quéré et al., 2016). Through the biological carbon pump, carbon fixed by photosynthesis in the upper ocean is exported to the ocean interior (Falkowski et al., 1998). In the North Atlantic Ocean, primary producers fix between 10 and 20 Gt of carbon annually, making this region an important sink of CO_2_ (Falkowski et al., 2000; Heinze et al., 2015). Estimates of carbon export for the North Atlantic (0–80°N) range from 0.5 to 2.7 Gt C/year, with an average of 1.3 Gt C/year (Sanders et al., 2014). The depth at which this carbon is remineralized determines whether organic carbon reaches the deep ocean and thus contributes to long‐term carbon sequestration. Kwon et al. (2009) showed that atmospheric carbon dioxide concentrations are highly sensitive to changes in this remineralization depth. In this modeling study, atmospheric CO_2_ concentrations fell by 20 to 27 ppm as a result of a simulated global increase of 24 m in remineralization depth (the depth at which 63% of sinking organic carbon is respired).

Despite their importance, global estimates of carbon export from the euphotic zone remain uncertain, ranging from 5 to >12 Pg C/year (Henson et al., 2011; Laws et al., 2000), and it is still unclear how strongly organic carbon fluxes are attenuated below the productive zone. Current estimates of carbon budgets for the mesopelagic zone (∼100–1,000 m) are not closed, with mesopelagic heterotrophic metabolic demands often exceeding supply by up to 2 orders of magnitude (Burd et al., 2010; Steinberg et al., 2008).

Traditionally, the majority of export by the biological carbon pump, and thus of carbon supply to the mesopelagic zone, is ascribed to sinking of large, fast‐sinking particles (Buesseler et al., 2007; Buesseler & Boyd, 2009), but recent findings have challenged this paradigm in two different ways. First, it is often assumed that small particles do not sink or sink so slowly that they are respired in the upper mesopelagic (Giering et al., 2014; Riley et al., 2012). However, several studies have found indications of a significant contribution to carbon export by small particles. For example, both Alonso‐Gonzalez et al. (2010) and Durkin et al. (2015) found evidence for small, slowly settling particles dominating the export size spectrum in sediment traps in the upper mesopelagic. Baker et al. (2017) showed that slow‐sinking particulate organic carbon (POC) export fluxes generally dominate over fast‐sinking fluxes in the upper mesopelagic and often increase with depth, likely resulting from fragmentation of larger, fast‐sinking particles.

Second, several alternative sinking pathways for organic carbon flux have been proposed. An additional seasonal flux of carbon to the mesopelagic zone could be provided by the mixed‐layer pump, exporting POC through sequential deepening and shoaling of the mixed layer in spring (e.g., Dall'Olmo et al., 2016; Gardner et al., 1995). The resulting detrainment of particle‐laden surface waters could cause a significant net carbon export flux, especially in high‐latitude areas where mixed layers are deep and temporally variable. Dall'Olmo and Mork (2014) observed deep fluxes of small‐particle POC with Bio‐Argo floats in the Norwegian Sea and suggested that these were partly driven by the seasonal mixed‐layer pump. Likewise, Erickson and Thompson (2018) showed that submesoscale instabilities of the mixed layer are a potential driver of export of fixed carbon, especially over a short time window in spring. Another alternative pathway was established by Omand et al. (2015), who showed that eddy‐driven subduction can sustain a downward carbon flux accounting for as much as 25% of the total (sinking) export flux, likely exporting small size classes of POC and dissolved organic carbon.

In summary, there is a lack of knowledge on the ecological and physical processes controlling the biological carbon pump, as well as uncertainty regarding its magnitude. A lack of agreement between various methodologies to estimate export, as well as scarce availability of in situ POC flux data, hinders a mechanistic understanding necessary for modeling and predictive purposes (Sanders et al., 2014). However, advances in autonomous tools, such as gliders and floats, have the potential to vastly expand our database of POC measurements. The use of optical backscatter sensors on gliders allows for the estimation of small‐particle (particle size range 0.2–20 μm) POC concentrations at high frequency (e.g., Briggs et al., 2011; Cetinić et al., 2012; Dall'Olmo & Mork, 2014) and can help clarify export pathways and patterns of vertical attenuation of POC in the water column.

In this study, we explore seasonal patterns of small‐particle carbon flux in the North Atlantic using a unique, yearlong data set of backscatter‐derived POC data at daily resolution, measured by Seagliders. In particular, we calculate POC concentration‐based transfer efficiency for small particles and implement a simple model to quantify export by the mixed‐layer pump, based on Dall'Olmo et al. (2016). We show that this export displays a strong seasonality, and our results imply that a net annual flux of small‐particle POC is sustained by the mixed‐layer pump.

## 2 Data and Methods

### 2.1 Study Site

**Figure 1:**
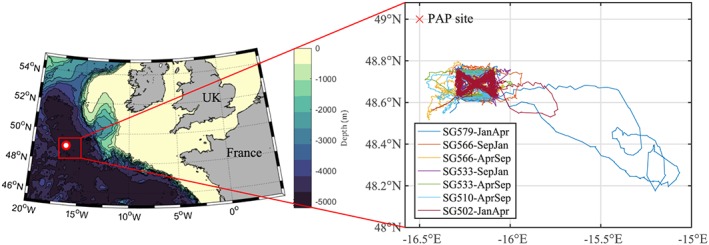
Location of the Porcupine Abyssal Plain (PAP) sustained observatory study site, with inset showing the trajectories followed by the different gliders.

This study was conducted around 40 km southeast of the Porcupine Abyssal Plain (PAP) sustained observatory site (49°N, 16.5°W) in the Northeast Atlantic (Figure 1). The PAP site is situated on the continental shelf at a water depth of ∼4,850 m (Hartman et al., 2010), in the open ocean between the subpolar and subtropical gyre. As it lies away from the continental slope, it experiences minimal effects of tides and strong permanent currents (Lampitt et al., 2010). The typical maximum depth of the winter mixed layer is around 350 m (Steinhoff et al., 2010) and the annually integrated primary productivity is ∼200 g C·m^−2^·year^−1^ (Lampitt et al., 2010).

### 2.2 Glider Mission

The Seaglider is an autonomous underwater vehicle that glides forward and, by controlling its buoyancy through an oil‐filled bladder, follows a vertical sawtooth pattern (Eriksen et al., 2001). As part of the NERC‐funded UK OSMOSIS (Ocean Surface Mixing, Ocean Submesoscale Interaction Study) project, five different Seagliders (SG579, SG501, SG533, SG566, and SG510) were deployed in three rotations of 3 to 5 months each, with at least two gliders deployed simultaneously (see Table 1), over the period September 2012 to September 2013. The gliders were programmed to follow a butterfly trajectory over a 20 × 20 km sampling area (Figure 1). Although occasional drifting was observed, 88% of the glider profiles were within the intended study area. All profiles, including those recorded during drifting, were included in the analysis, to ensure continuity of the time series. A full dive (to 1,000‐m depth) was completed in 4–5 hr, and the full sampling area was covered in ∼4 days. Each glider carried a CT sensor (measuring salinity and temperature), a Wetlabs Triplet ECOpuck (measuring chlorophyll‐*a* fluorescence, optical backscatter and colored dissolved organic matter), a hemispherical PAR sensor and a dissolved oxygen optode. To conserve battery power, the sampling depth and resolution of the ECOpuck sensors were continuously adjusted throughout the mission; sampling resolution was typically between 2‐ and 10‐m depth, with lower resolution for deeper profiles.

**Table 1:** Specifications of the Deployed Gliders

Glider	Deployment	Backscatter	SF^b^	DC^b^	DC
	period^a^	wavelength (nm)	(m^−1^ sr^−1^)		correction^c^
SG579	8 Jan to 23 Apr	532	9.003·10^6^	44	+1.01
SG566	1 Sep to 8 Jan	650	4.16·10^6^	35	+4.73
	19 Apr to 7 Sep				+5.47
SG533	1 Sep to 8 Jan	650	4.122·10^6^	45	−2.16
	3 Jun to 3 Aug				+0.49
SG510	19 Apr to 9 Jun	650	3.961·10^6^	49	−6.48
SG502	8 Jan to 23 Apr	700	3.011·10^6^	50	−0.34

a Dates are in 2013, except for SG566/SG533 rotation 1 (1 Sep 2012 to 8 Jan 2013).

b Scale factor (SF) and dark counts (DC) as supplied by manufacturer.

c Correction applied to manufacturer dark counts, from subtraction of the lowest 0.05 percentile over all *b*_bp_ data below 400 m.

Chlorophyll‐*a* (chl‐*a*) concentrations were determined from glider fluorescence data. First, manufacturer dark counts were reevaluated as the median value of fluorescence over the bottom 10 m of the fluorescence profiles. Quality control included a global range test, despiking, and a correction for daytime fluorescence quenching using glider optical backscatter data. The quenched part of the daytime profiles was corrected using the fluorescence‐to‐backscatter ratio from unquenched profiles collected close in time, following Sackmann (2008). Finally, chl‐*a* data were calibrated using in situ chl‐*a* samples. For more processing details of glider‐derived chlorophyll‐*a* concentrations, temperature, and salinity, see Rumyantseva (2016) and Damerell et al. (2016), respectively.

### 2.3 Optical Backscatter Data

The WETLabs BB2F ECOpuck measures the volume scattering function *β* at a centroid angle *θ* of 124° (revised from 117°, as reported in Sullivan et al. (2013)) at wavelengths of 532, 650, or 700 nm. The respective wavelengths and other specifications of the sensors deployed on each glider are shown in Table 1. Raw sensor counts were calibrated using manufacturer‐supplied scale factor (SF) and dark counts (DC) according to

(1) β(θ,λ)=[output−DC]·SF

The resulting volume scattering function *β*(*θ*,*λ*) includes both the particulate scattering signal and the scattering by pure seawater: *β* = *β*_*p*_+*β*_sw_ (e.g., Stramski et al., 2004; Zhang et al., 2009). The scattering by seawater *β*_sw_ was calculated using the model developed by Zhang et al. (2009), using the calibrated temperature and salinity glider data (for calibration procedures, see Damerell et al., 2016), a depolarization ratio of 0.039, the wavelength *λ* of the respective sensor and an in‐water centroid angle *θ* of the ECOpuck scattering sensor of 124°. After subtraction of *β*_sw_, the particulate volume scattering function *β*_*p*_ was converted to the particulate optical backscattering coefficient *b*_bp_, as

(2) $$b_{\text{bp}}=2\pi \;\chi_{p}\;\beta_{p}$$

(e.g., Briggs et al., 2011; Cetinić et al., 2012; Dall'Olmo & Mork, 2014), using a conversion coefficient *χ*_*p*_ of 1.076 (for *θ* = 124°; Sullivan et al., 2013).

Dark counts, representing the offset that is measured when no light reaches the sensor, are known to often differ from factory values (Sullivan et al., 2013). Therefore, manufacturer dark counts were reevaluated: the correction consisted of subtracting the 0.05 percentile over all data below 400 m from the total backscatter signal, for each glider individually. Resulting equivalent dark‐count corrections are given in Table 1. Single dives with a mean optical backscatter higher than 0.001 m^−1^ in deep water (>150 m) were removed, following Thomalla et al. (2015). These included two series of bad profiles with sudden, unrealistically high backscatter values extending over the full water column. Most likely, this was due to biofouling of the respective sensor. Data from SG579 and SG502 were then converted to a wavelength of 650 nm, assuming a *λ* ^−0.41^ spectral functionality for *b*_bp_ (Cetinić et al., 2012), to obtain a continuous *b*_bp_ time series. Up and down glider casts were averaged by day (midnight‐to‐midnight) and gridded onto a regular pressure grid (5‐m bins). A five‐point running median filter over depth was applied, followed by a seven‐point running mean filter, to eliminate spikes, assumed to be caused by aggregates (Briggs et al., 2011).

**Figure 2:**
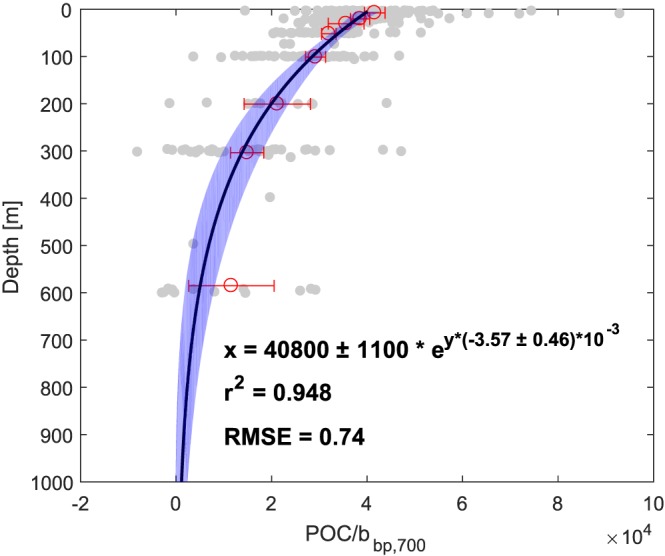
Determination of POC/b_bp_ slopes (λ = 700 nm) over depth from the NAB08 data set (data from Cetinić et al., 2012), with individual bottle data in gray, and slopes from a linear regression per depth bin in red (error bars represent the standard errors of the regressions). The black line is an exponential fit of the slopes per depth bin, weighted by the errors of the individual regressions (shaded blue area represents the confidence interval of the fit). POC = particulate organic carbon; RMSE = root‐mean‐square error.

Lastly, *b*_bp_ was converted to POC concentration using depth‐dependent *b*_bp_ to POC conversion factors, which we calculated from the NAB08 data set (Cetinić et al., 2012; Perry, 2011). As in our study, Cetinić et al. (2012) collected backscatter measurements in the North Atlantic, and it is, to our knowledge, the only study converting depth‐resolved backscatter to POC in the mesopelagic (up to 600 m), accounting for both small‐particle POC and dissolved organic carbon (DOC). The data set consists of 317 bottles for which POC and backscatter data were compared, roughly divided over eight bottle depths (between 10 and 97 bottles per depth across the entire data set). To determine the POC/*b*_bp_ slope over depth, we performed a linear regression (forced through zero) at each bottle depth, and then fitted an exponential function through these eight points, weighted by $\frac{1}{SD^{2}}$ of each linear regression (Figure 2). Slopes for the glider depth bins were calculated from the equation given in Figure 2, accounting for a difference in wavelength (700 nm for NAB08 versus 650 nm for our OSMOSIS data set) using *γ* = 0.41. Furthermore, there was a consistent offset of 1.33 × between the ship's backscatter sensors and the ECOpuck backscatter sensors of NAB08 (see Figure 3 in Briggs et al., 2011); we therefore multiplied our slopes by a further 1.33. Because the exponential fit underestimated the POC/*b*_bp_ slope at depth (> ∼500 m), we present our results as a lower boundary for POC (export). Reported errors on export values, resulting from the uncertainty in the POC/*b*_bp_ slope, are ±2*σ*.

### 2.4 Derived Parameters

#### 2.4.1 Mixed‐Layer and Euphotic Depths

Estimates of mixed‐layer depth were taken from Damerell et al. (2016) and based on the definition by de Boyer Montégut et al. (2004). Mixed‐layer depth (*z*_*m*_) was defined as the shallowest value of two criteria: (i) a change in temperature of 0.2 °C relative to the value at 10‐m depth; or (ii) a change in density of 0.03 kg/m^3^ relative to the value at 10‐m depth. In this way, we can find the depth of the mixed layer even when temperature and salinity vary with depth in a density‐compensating manner, and in cases where salinity, rather than temperature, causes density to vary with depth. Estimates of the depth of the euphotic zone (*z*_eu_) were calculated from Seaglider PAR data by fitting an exponential curve to daytime light profiles above 100 m (Rumyantseva et al., 2015). The euphotic depth was defined as the depth of 1% of surface irradiance. Because of glider surfacing maneuvers, the surface PAR data were not properly resolved; daily surface PAR values derived from MODIS Aqua satellite data, averaged over the sampling site, were used instead.

Lastly, the productive depth *z*_*p*_, computed as the deepest of the euphotic and the mixed‐layer depth, was used as the cutoff depth for the productive zone. The region from the surface to *z*_*p*_ defines the region in which phytoplankton can grow, and in which organic particles are therefore potentially produced. In February and March POC and chl‐*a* are not necessarily well mixed within the mixed layer, suggesting an active mixing layer is in place (sensu Brainerd & Gregg, 1995). The deviations between mixed and mixing layer occur during the end of winter and early spring and are short lasting (see also Rumyantseva, 2016). For consistency, we use *z*_*p*_ throughout our annual time series.

#### 2.4.2 Small‐Particle Transfer Efficiency

We define the small‐particle transfer efficiency (*R*_100_) as POC 100 m below *z*_*p*_ normalized by POC at *z*_*p*_. *R*_100_ is a measure of the efficiency with which small particles (and their carbon) are transferred from the productive layer into the mesopelagic, whether by the mixed‐layer pump, subduction, direct sinking, or aggregation, sinking, and subsequent disaggregation. It is analogous to the flux transfer efficiency *T*_100_ defined by Buesseler and Boyd (2009) as the carbon flux 100 m below *z*_eu_ normalized to flux at *z*_eu_. We chose to define *R*_100_ relative to *z*_*p*_, rather than *z*_eu_ for consistency with our export estimates, and we chose a ratio of concentrations in order to include the effects of POC transfer via subduction and the mixed‐layer pump, which are generally not included in *T*_100_.

#### 2.4.3 Mixed‐Layer Pump Model

To study the magnitude of the mixed‐layer pump, we implemented a simple model based on the methodology of Dall'Olmo et al. (2016), where we defined the export *E* at time *t* as the POC stock integrated over the change in the mixed‐layer depth between two subsequent time steps:

(3) $$E(t)=\;-\overset{z_{p}(t+1)}{\underset{z_{p}(t)}{\int }}\text{POC}(z,t)\;dz.$$

**Figure 3:**
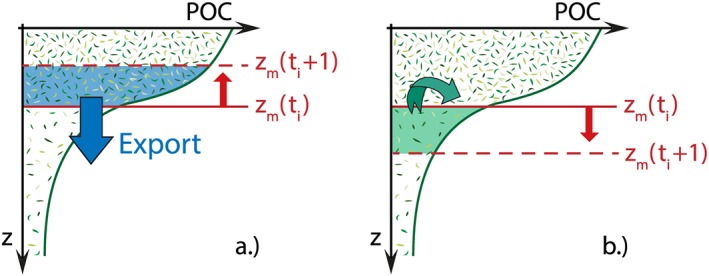
Working of the mixed‐layer pump: shoaling of the mixed‐layer depth z_m_ results in detrainment of particle‐laden surface waters, that is, export of particulate organic carbon (POC) stock from the mixed layer (a), whereas deepening of z_m_ results in entrainment of underlying waters with low POC concentration into the mixed layer (b).

In this formulation, whenever the mixed layer shoals (*z*_*p*_(*t*) > *z*_*p*_(*t* + 1)), export is positive and POC stock is detrained from the surface layer; when the mixed layer deepens, POC stock is entrained from deeper waters (Figure 3). Summing the daily export values, we calculated the total monthly and annual export by the mixed‐layer pump. To compare our results to the study by Dall'Olmo et al. (2016), we also computed the total export over the period defined in their study as the “seasonal” mixed‐layer pump: the period from the day on which maximum mixed‐layer depth (*z*_max_) is reached, until the start of summer stratification. The onset of summer stratification was defined as the first day on which the variation in mixed‐layer depth over 10 days was less than 20% of *z*_max_, for at least 30 days, following Dall'Olmo et al. (2016).

## 3 Results

### 3.1 POC Variability

**Figure 4:**
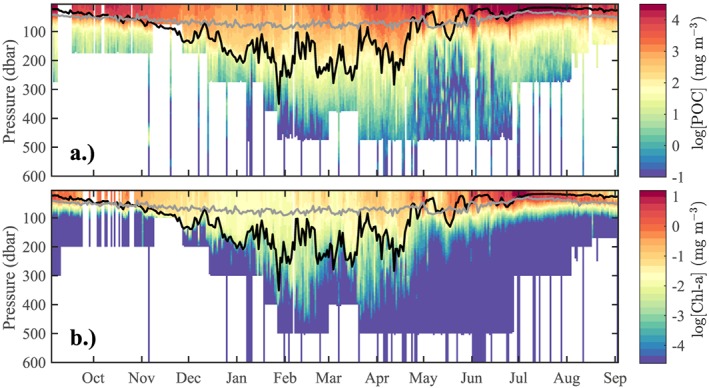
Vertical profiles (upper 600 m) of glider‐derived concentrations of particulate organic carbon (POC; a) and chlorophyll‐a (chl‐a; b) on a logarithmic scale, from September 2012 to September 2013. Depth of the euphotic zone z_eu_ and depth of the mixed layer z_m_ are indicated in gray and black, respectively. Black triangles in the upper plot represent glider turnover at the end of glider rotations 1 and 2 (see Table 1 for dates).

Within the mixed layer, POC concentration generally mirrored chl‐*a* concentration (Figure 4). From the start of the measurement period, both chl‐*a* and POC within the mixed layer were elevated and gradually decreased over October and November. Simultaneously, the mixed‐layer depth gradually deepened from a minimum of 22 m in September to just over 100 m at the end of November. Winter mixing was observed from December onwards, indicated by large variations in *z*_*m*_ (up to 90 m/day) until the end of April, with a maximum mixed‐layer depth of 350 m on 28 January. During this winter period, POC and chl‐*a* concentrations in the upper ocean were at their minimum levels.

From March onward, we observed a gradual accumulation of POC within the surface layer. In May, frequent shoaling of *z*_*m*_ above 100 m occurred; during shoaling periods, maximum surface POC values of up to 140 mg/m^3^ were reached, concurrently with intense peaks in chl‐*a*. One more deepening of *z*_*m*_ to 150 m was observed in late May, until final summer stratification set in at the end of June. Euphotic depth *z*_eu_ varied between 28 and 95 m, peaking in January when the mixed layer was deep, and reaching minimum values in the beginning of July, after the onset of the main chl‐*a* bloom. Shoaling of *z*_*m*_ above *z*_eu_ occurred occasionally from May 2013, until *z*_*m*_ remained below *z*_eu_ from the end of June onward.

Below the mixed‐layer depth, the coupling between POC and chl‐*a* concentrations disappeared, with chl‐*a* levels rapidly declining. In contrast, the POC signal penetrated deeper into the mesopelagic zone, and an increase in POC concentrations below *z*_*m*_ was observed in spring. We hypothesize that this seasonal flux is caused by the mixed‐layer pump and will assess this hypothesis further in section 3.3.

### 3.2 Small‐Particle Transfer Efficiency

**Figure 5:**
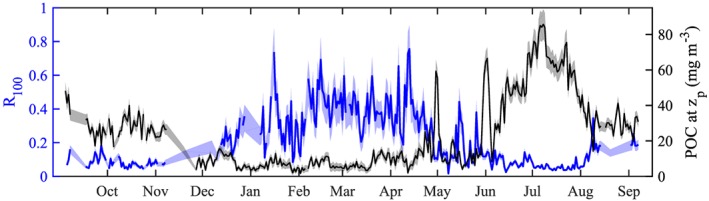
Small‐particle transfer efficiency R_100_ (blue) at 100 m below the depth of the productive zone z_p_. The black line shows the particulate organic carbon (POC) concentration at the productive depth. The light‐shaded areas represent the confidence intervals (±2σ) as a result of uncertainty in the b_bp_ to POC conversion.

Small‐particle transfer efficiency (*R*_100_) displayed a strong seasonality, peaking in spring (Figure 5). *R*_100_ was highest in February, with a mean of 0.42 ± 0.03 and was also relatively high (average values >0.3) in January, March, and May. Lowest values were reached in July (mean *R*_100_ of 0.05) and in June, October, and November *R*_100_ was also below 0.1. Over the period defined for the mixed‐layer pump (*t*_max_ until *t*_strat_), average transfer efficiency was 0.33 ± 0.03.

Strong high‐frequency variability in transfer efficiency was observed, reaching values up to 0.7 between January and April, indicating periods of very efficient transport of POC into the upper mesopelagic layer, relative to loss processes. From April to July, *R*_100_ increased during periods of mixed‐layer shoaling; this is another indication of POC export fluxes occurring concurrently with spring shoaling of the mixed layer, likely as a result of the mixed‐layer pump.

### 3.3 Mixed‐Layer Pump

**Figure 6:**
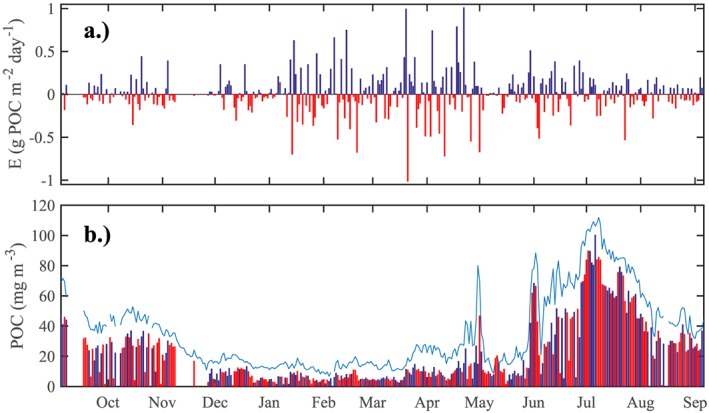
(a) Estimated daily export by the mixed‐layer pump. Positive export (blue) indicates detrainment of stock from the mixed layer, negative (red) indicates entrainment of particulate organic carbon (POC) stock from waters below. (b) Average POC concentration within the mixed layer (blue line) and average POC concentrations within the detrained and entrained waters (blue and red bars, respectively).

Daily export by the mixed‐layer pump ranged between −1.0 and 1.0 g POC/m^2^, with positive export, that is, detrainment or sinking, occurring on 163 out of 366 days (Figure 6a). Part of this export was counterbalanced by reentrainment of underlying waters, but summing over all recorded entrainment and detrainment events resulted in a net annual export by the mixed‐layer pump of 3.0 ± 0.6 g POC/m^2^.

The mean export of all recorded daily detrainment events was 0.17 g POC·m^−2^·day^−1^, whereas the mean of all entrainment events was −0.14 g POC·m^−2^·day^−1^. Both entrained and detrained POC concentrations were lower than the average mixed‐layer POC concentration (Figure 6b); however, detrained concentrations were generally higher than subsequent entrained concentrations, indicating that the surface mixed layer was more particle‐laden than the waters of the upper mesopelagic. Concentrations of mixed‐layer POC peaked near the end of April, after the onset of the main spring bloom (see Figure 4).

**Figure 7:**
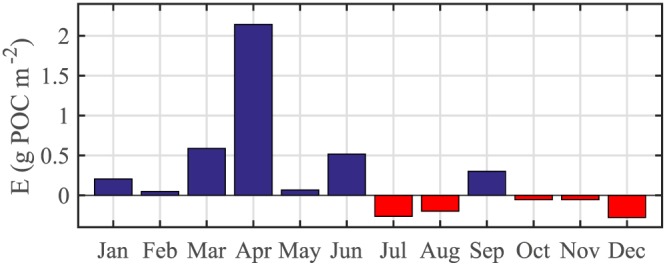
Net monthly export of particulate organic carbon (POC) by the mixed‐layer pump for the complete study period.

The mixed‐layer pump is mostly active in spring. A seasonal increase in POC flux is clearly visible below the mixed layer when plotting POC concentrations on a logarithmic scale (Figure 4). Summing only over the period from maximum mixed‐layer depth to the onset of summer stratification (t_max_ to t_strat_; 29 January until 30 May 2013) resulted in a seasonal export flux of 3.0 ± 0.6 g POC/m^2^, which is equal to our annual estimate. Figure 7 shows the monthly export for the full measurement period. Export peaked in April at 2.1 g POC·m^−2^ month^−1^, and was lowest in July and December, at −0.3 g POC·m^−2^·month^−1^.

In conclusion, intermittent shoaling of the mixed layer in spring, alternated with deepening events, effectively pumps carbon stocks to deeper waters. We could partly relate these variations in mixed‐layer depth to changes in forcing by wind and surface heat flux. For this analysis, we refer the reader to Supporting Information S1 (Kalnay et al., 1996; Thorpe, 2005; Wentz et al., 2015).

## 4 Discussion

### 4.1 Uncertainty Assessment

The POC estimates presented here are likely a lower boundary for POC concentrations, as they only include small‐particle contributions and because the fit of POC/*b*_bp_ slopes is a conservative estimate, especially at larger (>500 m) depths. The most significant source of uncertainty in this study is the conversion from *b*_bp_ to POC. Most published work on *b*_bp_ to POC conversion presents conversion slopes for the near surface. The NAB08 is the only available data set with bottle data into the mesopelagic, enabling us to account for changes in POC/*b*_bp_ slope with depth, and the only slope estimate that accounts for contribution from DOC. For comparison, the results from Stramski et al. (2008) could be used as an upper boundary for the slope; they measured a POC/*b*_bp_ slope of 53607 g POC/m^2^ at the surface, for a wavelength of 555 nm (not including upwelling data). This is a 25% increase compared to the surface slope applied in this study (assuming *b*_bp_∼*λ* ^−0.41^, Cetinić et al., 2012). Including this increase results in a final export of 3.8 POC·m^−2^·year^−1^. Compared to our estimate of 3.0 ± 0.6 POC·m^−2^·year^−1^, this confirms our conclusion that we present here a lower boundary for small‐particle POC concentrations.

Another source of uncertainty lies in our method of dark‐count reevaluation, necessary since dark counts of backscatter sensors are known to often differ from factory values (Boss et al., 2008; Cetinić et al., 2009). Accuracy in future work could be improved by taking in situ measurements of sensor dark counts. Furthermore, conversion of the measured *b*_bp_ signal to a different wavelength adds uncertainty to our estimates, as the spectral slope coefficient is not strictly constant, but decreases with larger particle size (Wozniak & Stramski, 2004).

To present average POC values over the sampling site, we chose to grid our data to daily averages. With two gliders present simultaneously, all corners of the 20 × 20‐km sampling square were reached in about 2 days. A higher temporal resolution could therefore result in bias from spatial variability, as single time steps then represent a smaller area of the sampling site. The effect of temporal resolution for the backscatter data was investigated; a change in temporal resolution from daily to 6‐hourly increased the annual export estimate to 5.6 g POC/m^2^. As outlined above, we believe this increase mostly reflects increased spatial patchiness. However, we do recognize that the daily gridding removes any subdaily variations in mixed‐layer depth. Again, this emphasizes that we present here a lower boundary for annual POC export by the mixed‐layer pump.

Lastly, there were some short (1–6 days) data gaps where no glider backscatter profiles were available, and one gap of 10 days where backscatter profiles did not extend all the way to the depth of the mixed layer. As the longer data gaps occurred mostly during mixed‐layer deepening (September and November 2012), this could lead to a positive bias in our annual export estimate. Interpolation (spline) of these gaps in the POC time series leads to an annual export of 2.7 g POC·m^−2^·year^−1^, which is indeed lower than our estimate of 3.0 ± 0.6 g POC·m^−2^·year^−1^.

To conclude, there is both an additional uncertainty in the upper boundary for export due to the surface POC/*b*_bp_ slope and in the lower boundary due to data gaps. We incorporate this as an additional ±0.3 marge in our error estimate, leading to a final export of 3.0 ± 0.9 g POC·m^−2^·year^−1^.

### 4.2 Seasonality in Transfer Efficiency

Using glider‐derived estimates of POC in the upper mesopelagic, we showed that there is a strong seasonality in carbon export dynamics. First, seasonal patterns were detected in small‐particle transfer efficiency. Originally, transfer efficiency was defined by Buesseler and Boyd (2009) as normalized to the depth of the euphotic zone. However, for consistency in our results, we computed transfer efficiency relative to *z*_*p*_, so that it represents a measure of particle attenuation in the first 100 m below the productive zone. Our results indicate highly efficient transfer of POC through the upper mesopelagic in winter (peak *R*_100_> 0.7). In contrast, after mixed‐layer shoaling in spring, particle attenuation increases (*R*_100_<0.2), implying a weak efficiency of the small‐particle carbon pumps. The transition period between these two regimes occurs in April and May. Intermittent shoaling of the mixed layer results in short bursts of phytoplankton growth while transfer efficiency is still relatively high, which has important implications for the fate of particles exported by the mixed‐layer pump (section 4.4). This is in agreement with Erickson and Thompson (2018), who suggest that physically driven export (due to submesoscale instabilities) in our study site occurs in a short window in spring.

The peaks we observe in *R*_100_ in late spring are similar to a *T*_100_ of 80% reported by Buesseler and Boyd (2009) for the NABE in April/May 1989. Marsay et al. (2015) deployed PELAGRA sediment traps at the PAP site in July/August 2009. Using a power law for the attenuation of the flux *f* between depths *z*_0_ and $z,f_{z}=f_{z_{0}}{\left(\frac{z}{z_{0}}\right)}^{-b}$ (Martin et al., 1987), they found a coefficient of flux attenuation *b* of 0.7 ± 0.09. Our mean transfer efficiency over July and August, with a mean productive depth of 40 m, is 0.08 ± 0.2, resulting in a coefficient *b* of 0.57 ± 0.03. Although our small POC concentration ratio *R*_100_ is not equivalent to the flux ratio *T*_100_, it is interesting that our estimate of POC decrease is in quite good agreement with the estimates of flux decrease presented by Marsay et al. (2015).

While the observed seasonality in small‐particle transfer efficiency is likely driven in large part by changes in stratification, it may also reflect changes in particle consumption. For example, increased summertime zooplankton abundance and metabolism might reduce particle export through grazing activities (Cavan et al., 2017). Alternatively, the microbial community could display an increased abundance or increased remineralization rates due to increased temperatures observed in the upper mesopelagic in summer (Rivkin & Legendre, 2001). Further studies of the microbial and planktonic community are necessary to address any of these hypotheses.

### 4.3 Significant Export by the Mixed‐Layer Pump

Our final estimate for the total annual export flux by the mixed‐layer pump is 3.0 ± 0.9 g POC·m^−2^·year^−1^. The export estimate summed over the spring period only (end of January to middle of May) was the same as the annual estimate: 3.0 g POC/m^2^. This is consistent with the expectation that the mixed‐layer pump is truly a seasonal phenomenon, with entrainment and detrainment over the rest of the year canceling out. During summer, a subsurface chl‐*a* bloom developed below the productive depth, likely causing net POC entrainment into the productive layer and suggesting that our productive layer estimate may be too shallow during this period. In autumn, POC entrainment is most likely linked to deepening of the mixed‐layer.

Dall'Olmo et al. (2016) present a satellite‐based estimate of 8–10 g POC·m^−2^ year^−1^ for the seasonal mixed‐layer pump in the region around the PAP site, which is 2 to 3 times higher than our estimate. This difference could be due to differences in methodology; unlike Dall'Olmo et al. (2016), we do not assume a homogenous POC concentration over the mixed layer but use direct measurements of POC stocks both in the detrained and entrained waters. On the other hand, as discussed in section 4.1, our estimates are a lower boundary for POC export and are therefore not incompatible with the study by Dall'Olmo et al. (2016).

Total annual POC export at the PAP site specifically has been estimated at 15, 23, and 48 g C·m^−2^·year^−1^ from various satellite‐derived empirical algorithms (Dunne et al., 2005; Henson et al., 2011; Siegel et al., 2014). Compared to these estimates, the mixed‐layer pump would contribute between 5% and 25% of total annual export. However, the in situ estimates of export which are used to construct the empirical satellite algorithms are based on a combination of sediment trap and radioisotope (^234^Th) data, which may not account for small particles (Buesseler et al., 2007). Seasonal export by the mixed‐layer pump could therefore also (partially) be occurring in addition to the above estimates.

### 4.4 The Role of Small Particles

The backscatter sensors on the gliders observe particles in the range of 0.2–20 μm (Dall'Olmo et al., 2012; Dall'Olmo & Mork, 2014), that is, relatively small and presumably slow sinking. Therefore, the mixed‐layer pump may be an important pathway for both small particles and dissolved organic carbon to depth and can provide an additional carbon flux that may not have been previously accounted for in export estimates. As such, the mixed‐layer pump may contribute to closing the gap in current estimates of mesopelagic carbon budgets (Burd et al., 2010; Steinberg et al., 2008).

Recent studies have attributed a dominant role to small and/or slowly sinking particles in export from the surface (Alonso‐Gonzalez et al., 2010; Baker et al., 2017; Riley et al., 2012). The low settling speed and organic content of these small particles imply that they are mostly remineralized before reaching the abyssal ocean. However, in this study, most export took place in the month of April, and small‐particle transfer efficiency is relatively high. Indeed, we observed substantial elevated POC in the winter well below the deepest observed mixed‐layer depth. This implies that the mixed‐layer pump, perhaps in combination with further subduction and/or sinking, may be an efficient mechanism for exporting particles deeper into the mesopelagic, possibly contributing to deep carbon sequestration fluxes. This would be in agreement with results of Dall'Olmo and Mork (2014), who observed small particles at depths up to 900 m, which is deep enough to escape reentrainment by the following mixed‐layer deepening.

Observed small‐particle POC concentrations at depth may also result from midwater fragmentation of large aggregates, sinking from the surface layers (Burd & Jackson, 2009; Giering et al., 2016). Particles in the mixed layer can aggregate depending on particle concentration, particle size spectrum and mixed‐layer depth (Jackson & Lochmann, 1992; Jackson & Burd, 1998), and “stickiness” from polysaccharides, that is, transparent exopolymer particles (Passow et al., 1994). When newly formed aggregates reach a critical density, they sink out of the mixed layer and can subsequently disaggregate and become remineralized at depth (Burd & Jackson, 2009). Several recent studies have found evidence for an increasing contribution of small particles to flux with depth (Baker et al., 2017; Durkin et al., 2015), implying that midwater fragmentation is causing in situ generation of slow‐sinking POC below the mixed layer. Lastly, Giering et al. (2016) suggested that increased aggregate formation initiated by the passage of a storm could also be a significant contributor to export, with a magnitude similar to that of the mixed‐layer pump.

## 5 Conclusions and Future Work

We investigated a full annual cycle of POC export variability over the upper mesopelagic zone, derived from optical backscatter sensors on gliders. Our results imply that remineralization of particles in the mesopelagic displays a strong seasonality, with shallower remineralization occurring in late spring and summer, when POC concentrations are highest. Besides, we found evidence for a significant carbon supply to the mesopelagic zone by small particles through the mixed‐layer pump. On an annual timescale, the mixed‐layer pump in the PAP site was estimated to contribute between 5% and 25% of total POC export.

An important question that remains to be addressed in future studies concerns the fate of the exported particles. How the POC flux is attenuated below the productive zone determines whether fluxes contribute to long‐term carbon sequestration or sustain a carbon flux to the mesopelagic but remains an open question. Furthermore, research efforts should continue to improve and evaluate methodologies to estimate sinking fluxes from autonomous tools, since autonomous vehicles provide high‐resolution, sustained observations over time spans of months. Lastly, the effects of physical forcing on export by the mixed‐layer pump (see supporting information) could be further investigated through model simulations.

## Supporting information



Supporting Information S1
