# Supplementary material for: High‐Frequency Variability of Small‐Particle Carbon Export Flux in the Northeast Atlantic
Source: Global Biogeochem Cycles. 2018 Dec 18;32(12):1803–14. doi: 10.1029/2018GB005963 (PMC6472636; doi:10.1029/2018GB005963)
Supplement: Supplementary file 1 — Supporting Information S1 [file GBC-32-1803-s001.pdf]

Supporting Information for "High-frequency variability of small-particle carbon export flux in the Northeast Atlantic"

Roséanne Bol[1,2], Stephanie A. Henson[3], Anna Rumyantseva[1]and Nathan Briggs[3]

[1]University of Southampton, United Kingdom
[2]Now at: NIOZ Royal Netherlands Institute for Sea Research and Utrecht University, Den Hoorn, Texel, The Netherlands
[3]National Oceanography Centre Southampton, United Kingdom

Contents

1. Text S1: Physical forcing
2. Figure S1: export, wind speed and heat flux

Text S1: Physical forcing

Mixing regimes in the ocean surface layer are characterized either as wind or as convective mixing regimes [*Thorpe*, 2005]. Strong surface wind forcing may remove stratification and deepen the mixed layer; high POC export may be induced especially by strong storms passing later in spring, when particles have started accumulating within the mixed layer. In contrast, increased convective mixing is characterised by increased negative heat flux, enhancing turbulence through increased ocean to air heat loss.

Satellite time series of wind speed and heat flux were obtained to explore potential physical forcing mechanisms of the mixed-layer pump. Wind speeds were taken from the Cross-Calibrated Multi-Platform (CCMP) VAM Analysis Wind Fields dataset [*Wentz et al.*, 2015] on the grid point nearest to the OSMOSIS study site (-16.125°E; 48.625°N). This dataset consists of a 0.25°x0.25°, 6-hourly wind speed field at 10m above sea level. Net surface heat flux (NSHF) data were extracted from the NCEP/NOAA dataset with a 1.875°x1.875°, daily resolution [*Kalnay et al.*, 1996], again at the nearest grid point (-16.8750°E; 48.5705°N). Net surface heat flux was computed as the sum of sensible heat flux, latent heat flux, shortwave radiation and longwave radiation, where positive heat flux was defined as heat entering the ocean at the air-sea interface.

Time series of wind speed and net surface heat flux could provide clues to the forcing of the variations in mixed-layer depth that drive export events. Figure S1 shows these time series for the two months during which most export was observed (March and April 2013). Wind speed (figure S1b) was highly variable, varying between 2 and 19 m s^{-1} and peaking above 7 on the Beaufort scale (above 13.9 m s^{-1}) in 6 separate storm events during this 8-week period. Net surface heat flux (NSHF; figure S1c) during this period varied between approximately -300 and +120 W m^{-2}, and was negative most of the time, indicating net heat loss from the ocean. Brief periods of positive NSHF became prolonged and more frequent as spring progressed.

Two examples of ephemeral stratification events with relatively high export are highlighted in figure S1. In both cases, wind speed dropped substantially after a storm event, simultaneously with a switch from negative to positive NSHF. The resulting

Corresponding author: Roséanne Bol, roos.bol@nioz.nl

large shoaling in the mixed-layer depth (over 150m within 7 days) led to a net export of 1.3 and 2.3 g POC m^{-2} over the course of 1 week, respectively. As subsequent mixed-layer deepening occurred more gradually than the preceding shoaling, it is likely that (part of) the exported particles penetrated into the mesopelagic instead of being re-entrained into the mixed layer.

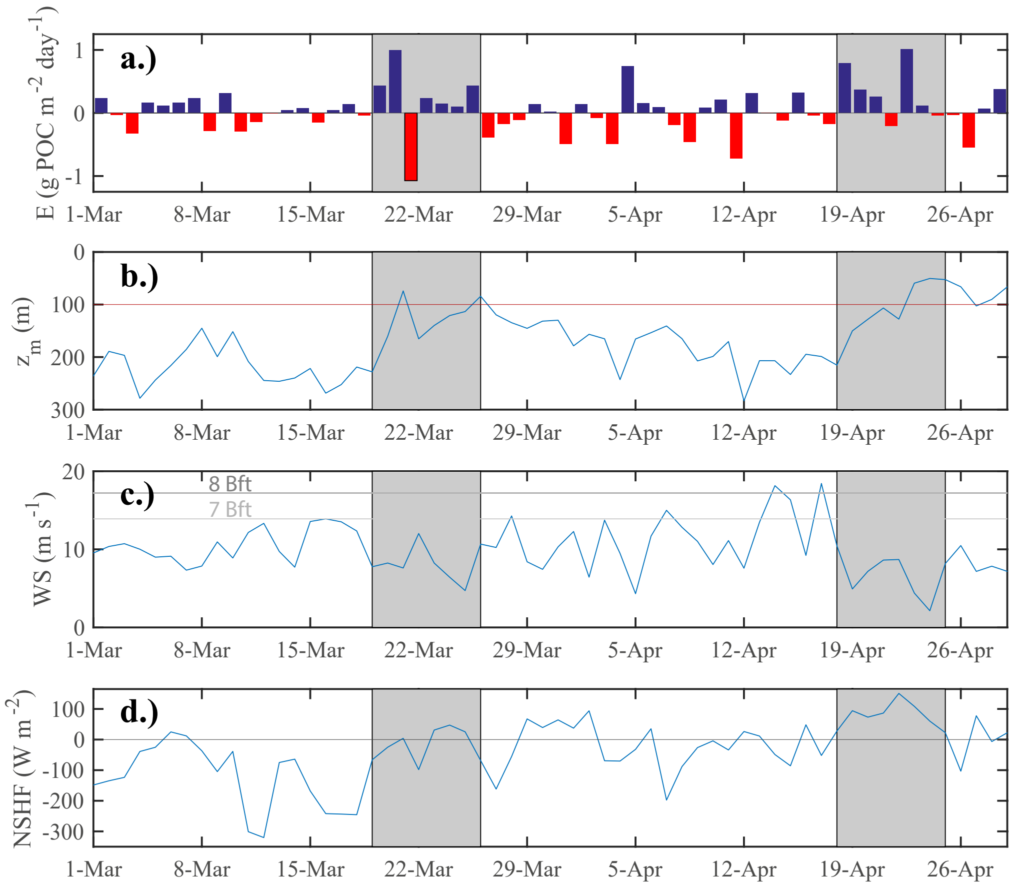

Figure S1.

Part of the export timeseries (a.) and corresponding mixed-layer depth (b.), wind speed (c.) and net surface heat flux (ocean-to-air; d.). Two ephemeral stratification events with a strong decrease in z_m are highlighted in grey; these events occur simultaneously with a decrease in wind speed and a switch from negative to positive heat flux.

References

Kalnay, E., M. Kanamitsu, R. Kistler, W. Collins, D. Deaven, L. Gandin, M. Iredell, S. Saha, G. White, J. Woollen, Y. Zhu, A. Leetmaa, and R. Reynolds (1996), The NCEP/NCAR 40-year reanalysis project, *Bulletin of the American Meteorological Society*, *72*, 437–470.

Thorpe, S. (2005), *The turbulent ocean.*, Cambridge: Cambridge University Press.

Wentz, F. J., J. Scott, R. Hoffman, M. Leidner, R. Atlas, and J. Ardizzone (2015), Remote Sensing Systems Cross-Calibrated Multi-Platform (CCMP) 6-hourly ocean vector wind analysis product on 0.25 deg grid, Version 2.0, [04-09-2012 to 07-09-2013]., *Remote Sensing Systems, Santa Rosa, CA.*, *Available online at www.remss.com/measurements/ccmp*, [Accessed 07 004 2017].
